# Supplementary material for: CRISPAltRations: a validated cloud-based approach for interrogation of double-strand break repair mediated by CRISPR genome editing
Source: Mol Ther Methods Clin Dev. 2021 Apr 2;21:478–91. doi: 10.1016/j.omtm.2021.03.024 (PMC8082044; doi:10.1016/j.omtm.2021.03.024)
Supplement: Document S1. Figures S1–S14 [file mmc1.pdf]

OMTM, Volume *21*

Supplemental information

CRISPAltRations: a validated cloud-based approach for interrogation of double-strand break repair mediated by CRISPR genome editing

Gavin Kurgan, Rolf Turk, Heng Li, Nathan Roberts, Garrett R. Rettig, Ashley M. Jacobi, Lauren Tso, Morgan Sturgeon, Massimo Mertens, Roel Noten, Kurt Florus, Mark A. Behlke, Yu Wang, and Matthew S. McNeill

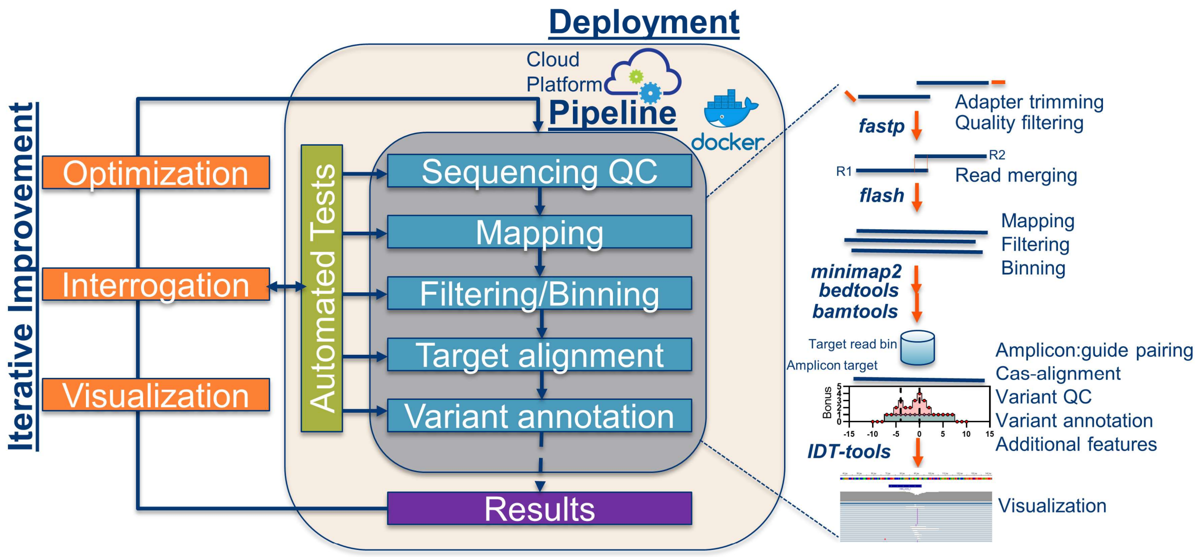

Figure S1. Development framework for CRISPAltRations. CRISPAltRations was architected such that each step of the pipeline (grey box) is containerized and deployed within the cloud to enable highly scalable batch processing (tan box). Briefly, the pipeline goes through a number of processing steps (blue boxes) to transform demultiplexed reads to results that quantify editing events after CRISPR genome editing (purple box) which can be viewed and stored in the cloud or downloaded locally. To improve CRISPAltRations, we used iterative improvement (orange boxes) to iterate through a process in which we manually inspected and interrogated experimental results to build tests (green box) which ensure stability, coverage of different experimental use-cases, and allowed us to optimize the software tool.

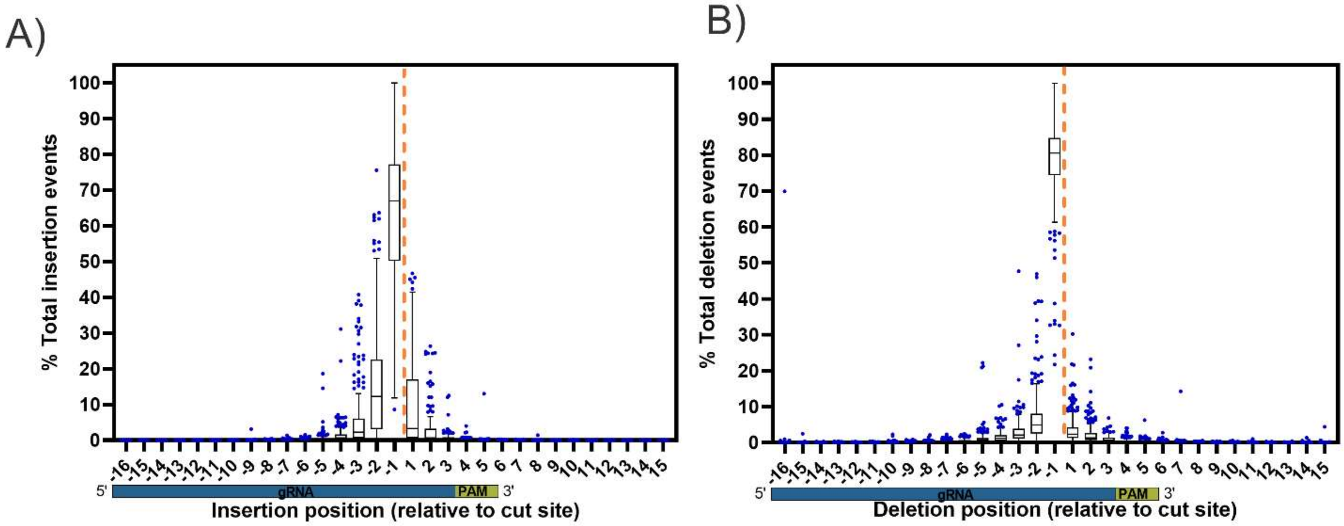

Figure S2. Characterization of Cas9-specific indel profiles using the standard Needleman-Wunsch alignment algorithm (Software iteration #1). Tukey box and whisker plot of A) insertion position, and B) deletion position profiles relative to the cut site (orange dashed line) of Alt-R S.p. Cas9 V3 (n=273 guides) editing events delivered via ribonucleoprotein nucleofection into Jurkat cells analyzed using software iteration #1.

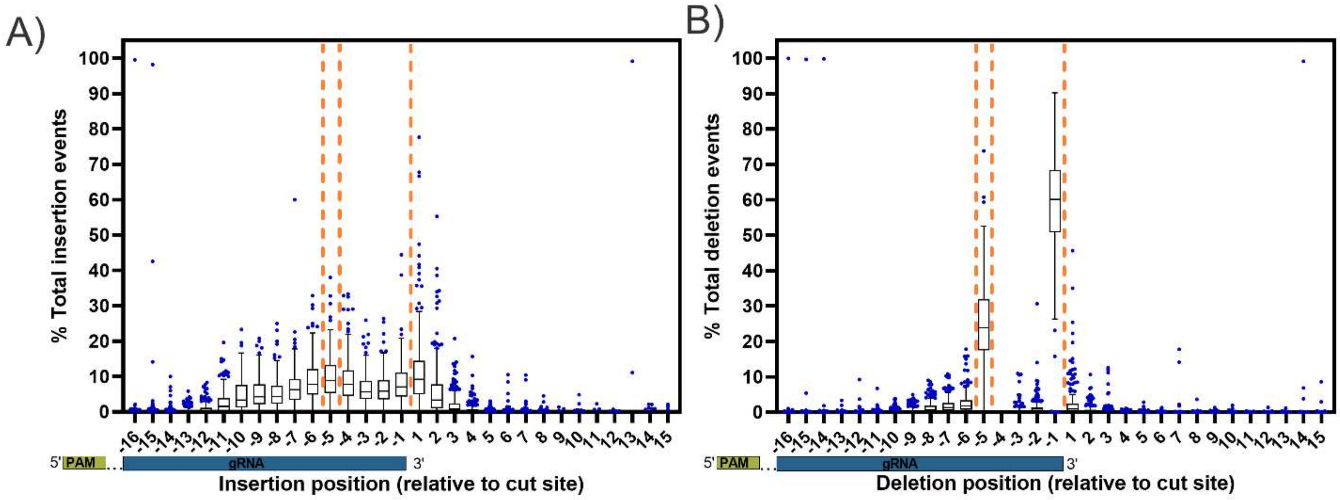

Figure S3. Characterization of Cas12a-specific indel profiles using the standard Needleman-Wunsch alignment algorithm (Software iteration #1). Tukey box and whisker plot of A) insertion position, and B) deletion position profiles relative to the putative nick sites (orange dashed line) of Alt-R A.s. Cas12a Ultra V3 (n=243 guides) editing events delivered via ribonucleoprotein electroporation into Jurkat cells analyzed using software iteration #1.

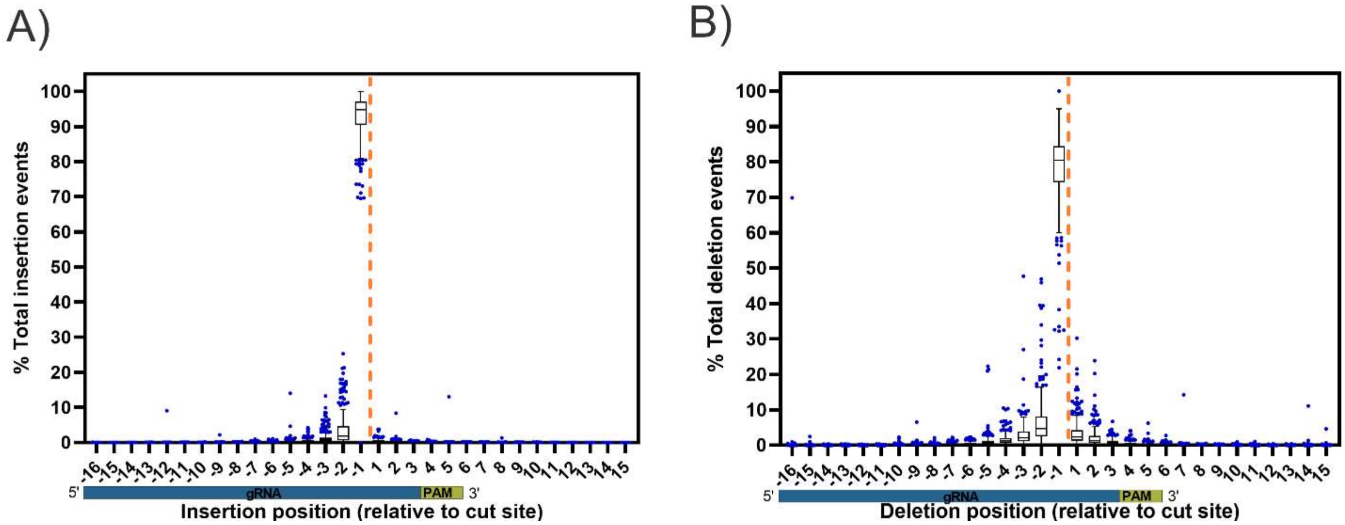

Figure S4. Characterization of Cas9-specific indel profiles for using psnw alignment algorithm with a single cut site bonus (Software iteration #2). Tukey box and whisker plot of A) insertion position, B) deletion position profiles relative to the cut site (orange dashed line) of Alt-R S.p. Cas9 V3 (n=273 guides) editing events delivered via ribonucleoprotein electroporation into Jurkat cells analyzed using software iteration #2.

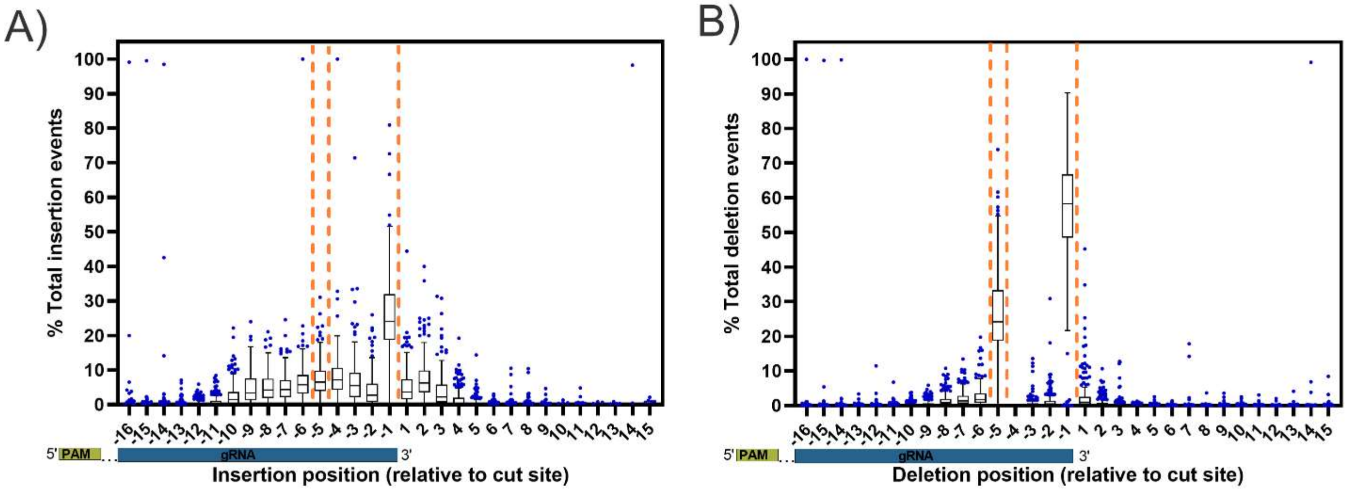

Figure S5. Characterization of Cas12a-specific indel profiles using psnw with a single PAM distal cut site bonus (Software iteration #2). Tukey box and whisker plot of A) insertion position, and B) deletion position profiles relative to the putative nick sites (orange dashed line) of Alt-R A.s. Cas12a Ultra V3 (n=243 guides) editing events delivered via ribonucleoprotein electroporation into Jurkat cells analyzed using software iteration #2.

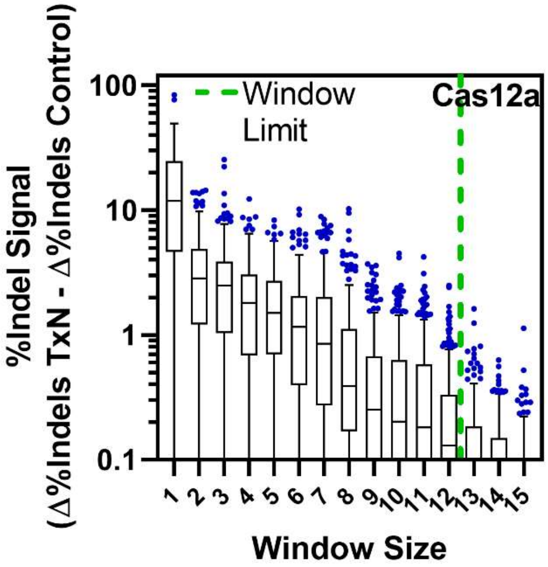

Figure S6. Window optimization AsCas12a centered at the PAM-distal nick site. An optimal window size (green dashed line) for annotating variants was selected for Alt-R A.s. Cas12a Ultra V3 editing in Jurkat cells at which median indel signal differences between treatment and control samples < 0.1%, with the window centered at the PAM-distal nick site.

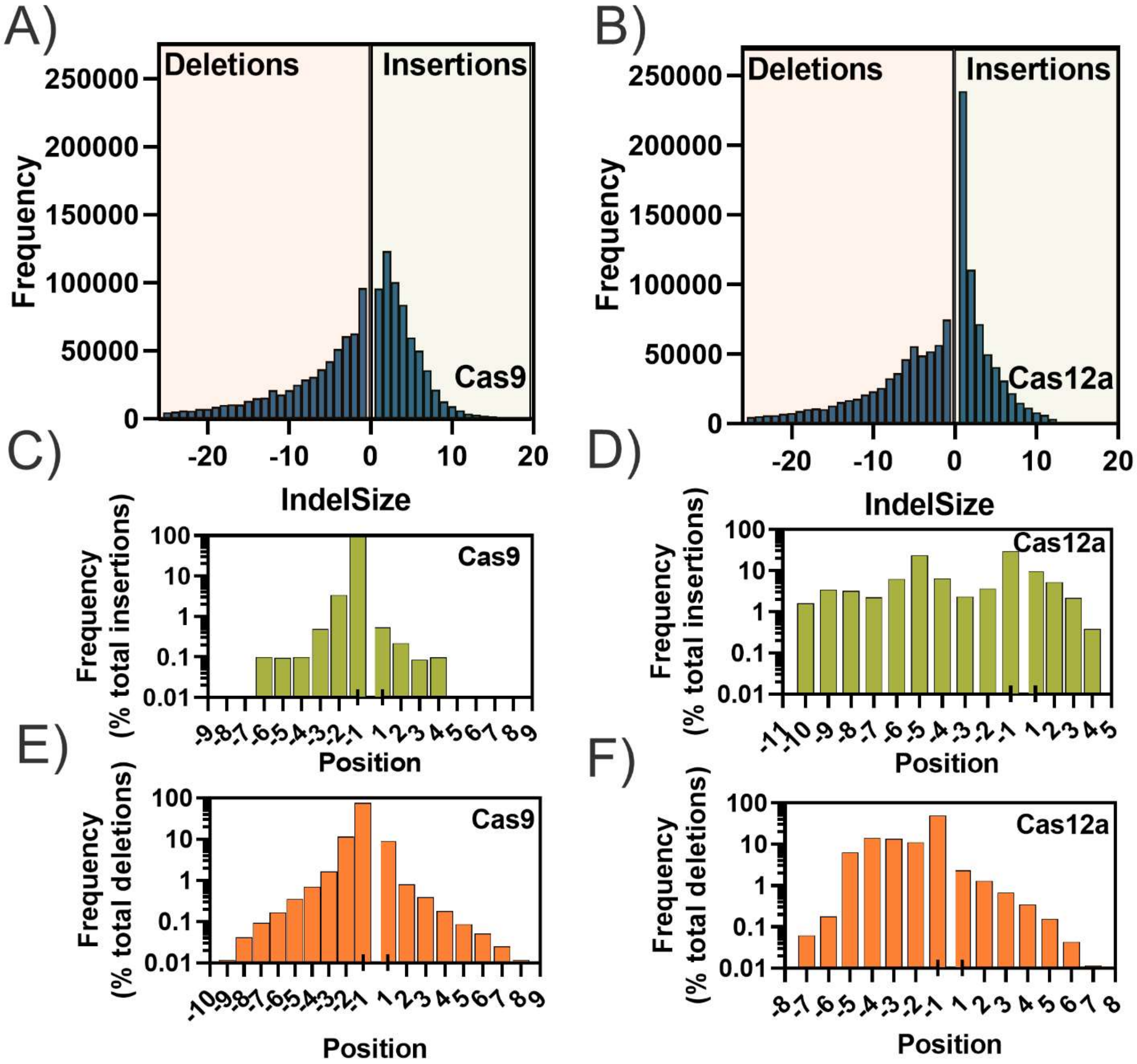

Figure S7. Synthetic on/off-target dataset used for pipeline validation. Characterization of synthetic CRISPR NGS on/off-target benchmarking data A-B) indel sizes, C-D) insertion positions, and E-F) deletion positions, all modeled based on experimental Alt-R S.p. Cas9 V3 or Alt-R A.s. Cas12a Ultra V3 editing data in Jurkat cells.

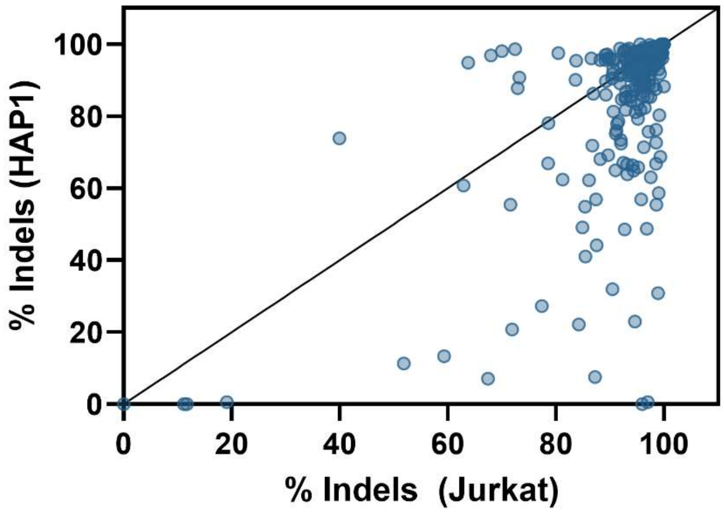

Figure S8. SpCas9 editing in HAP1 and Jurkat Cells. Quantification and comparison of indel editing by CRISPAltRations in HAP1 and Jurkat cell lines with Alt-R S.p. Cas9 V3 (n=273 unique gRNAs).

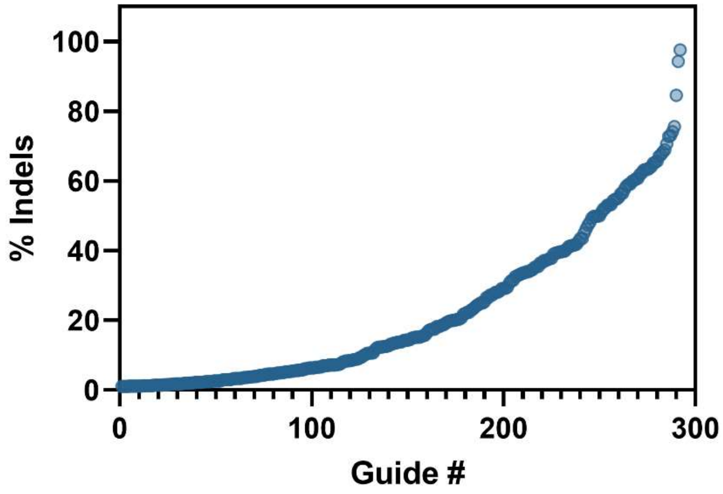

Figure S9. AsCas12a editing efficiency in Jurkat. Quantification of editing by CRISPAltRations in Jurkat delivered Alt-R A.s. Cas12a Ultra V3 (n=243 unique gRNAs)

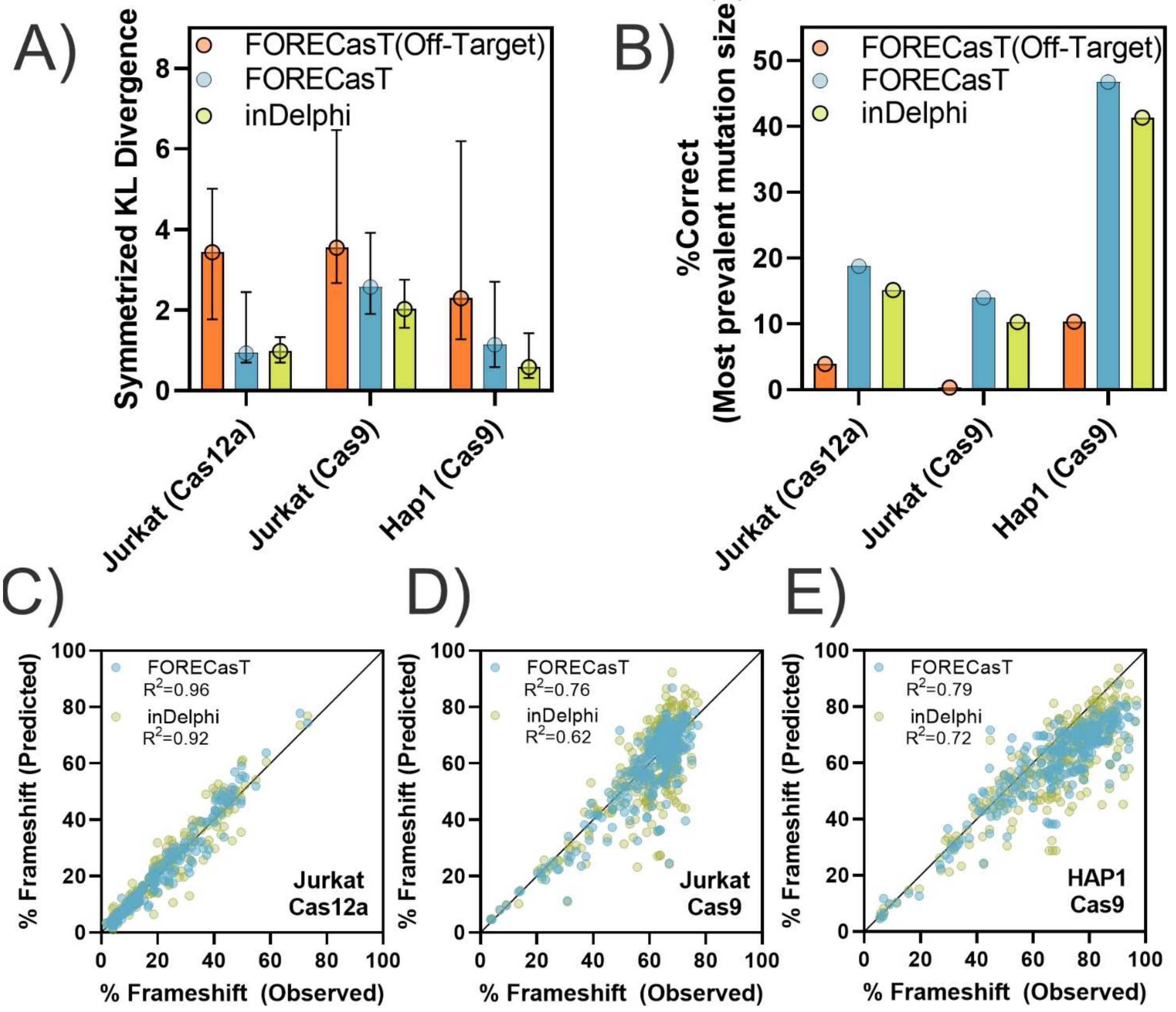

Figure S10. Performance of *in-silico* mutation profile prediction tools. FORECast and inDelphi were evaluated for the ability to predict mutation size distributions similar to what was observed Jurkat/HAP1 cells treated with Alt-R S.p. Cas9 V3 Cas9 or Alt-R A.s. Cas12a Ultra V3 by measuring A) symmetrized KL divergence between observed and predicted profiles (median ± IQR) and B) the mean accuracy predicting the most prevalent mutation. Linear regression was performed using predicted vs observed frameshift frequencies for C) Jurkat + Cas12a D) Jurkat + Cas9 and E) HAP1 + Cas9 treated cells with a line of identity (solid black line) displayed at y = x.

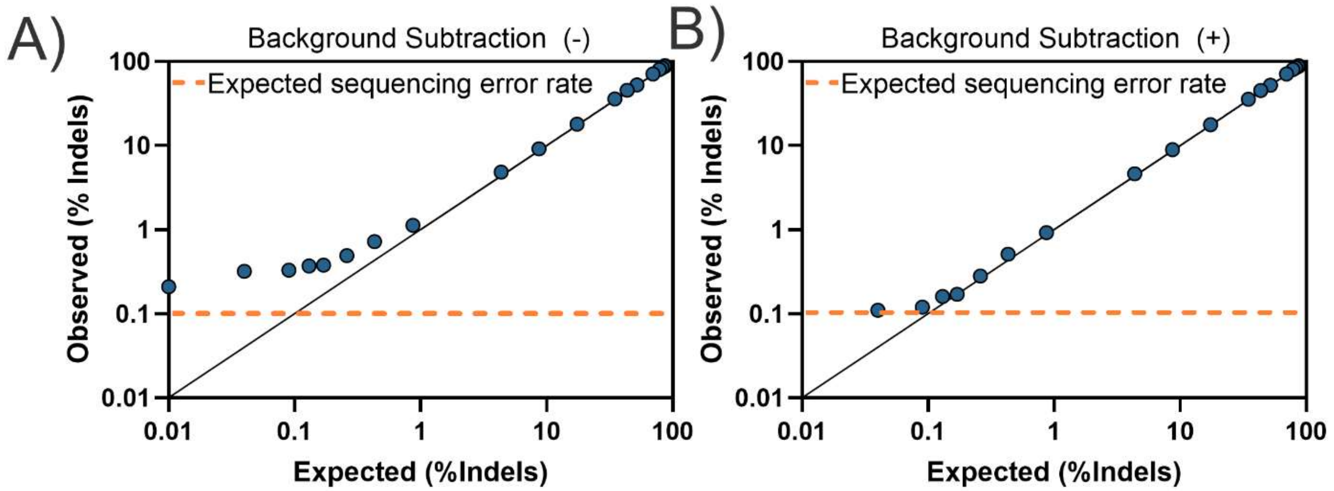

Figure S11. Pipeline indel detection sensitivity. Pipeline indel detection concordance (black line) with a titrated mixture of gBlocks with known concentrations of indels for an HPRT target (>40,000 reads per sample) sequenced with MiSeq v3 chemistry A) before and B) after a simple background subtraction, performed by subtracting the percent indels observed in an unmodified gBlock control from all samples.

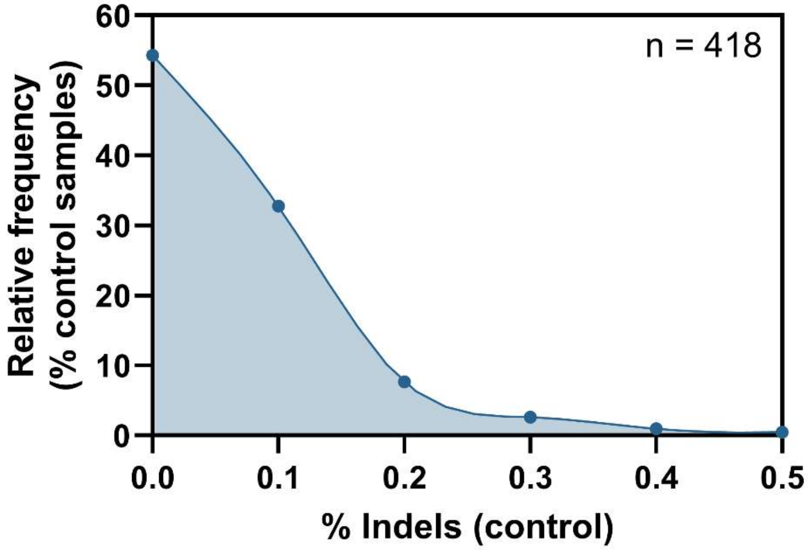

Figure S12. Evaluation of indel background noise in experiments. Relative frequency of unedited control samples with variable indel editing signal (binned in 0.1% intervals) for the same genomic targets from Jurkat (n=260) and HAP1 (n=158) cell lines in two separate experiments with high read depth (> 10,000 read pairs).

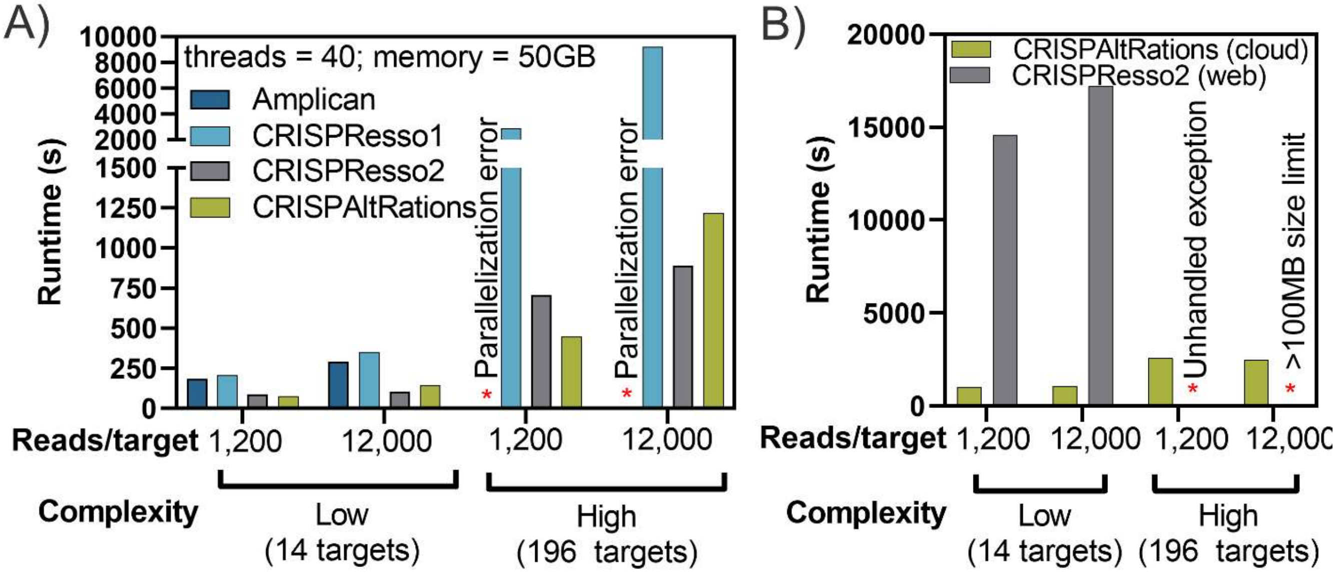

Figure S13. Pipeline runtime requirements. All multiplex compatible pipelines were ran against synthetic multiplex on/off-target datasets with 14 or 196 targets at varying read depth. Runtime in seconds was recorded for A) Command line interface and B) Web UI runs. Runs that failed submission or analysis are indicated (*).

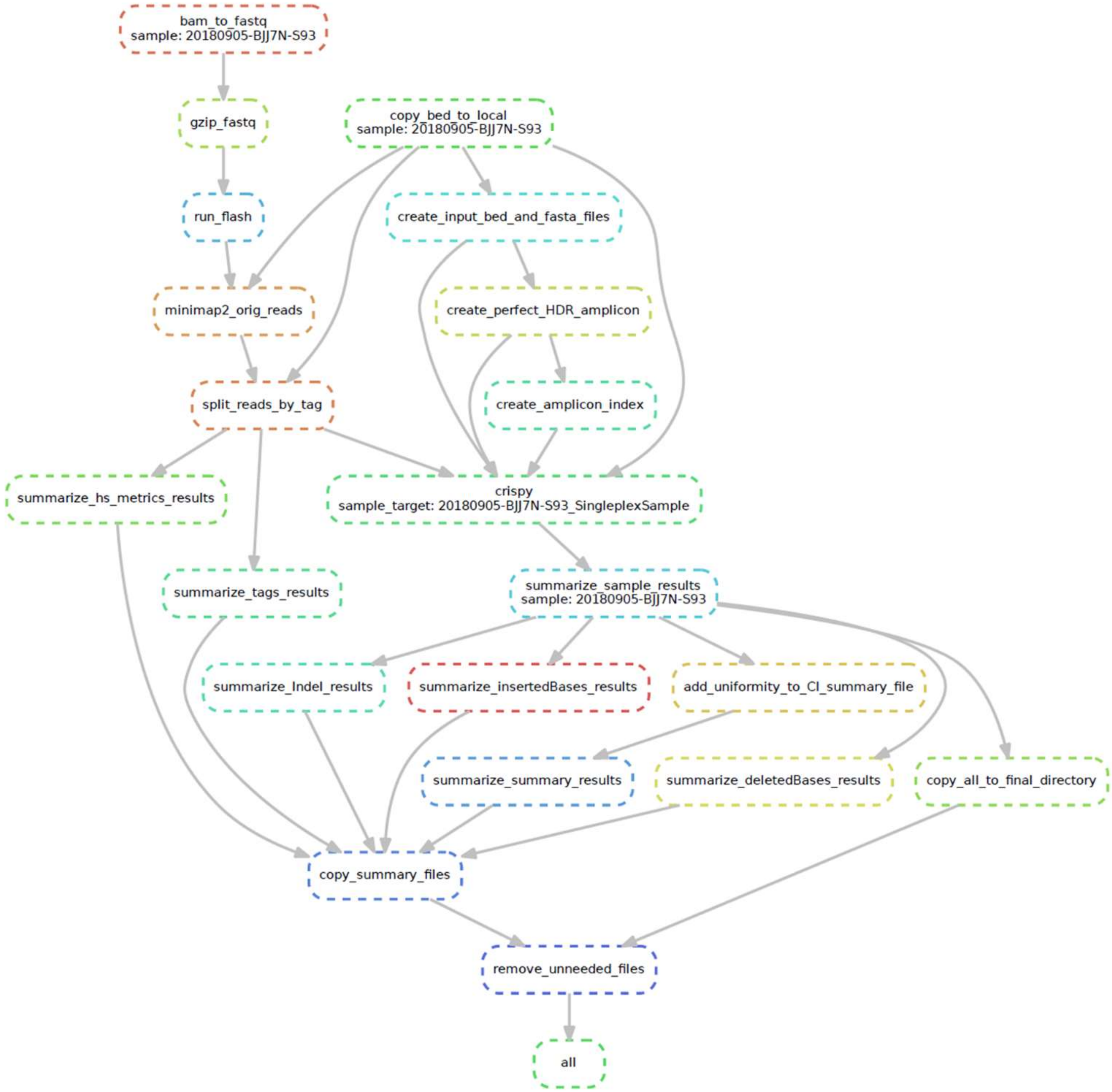

Figure S14. Directed Acyclic Graph (DAG) of the CRISPAltRations workflow. A single example sample (20180905-BJJ7N-S93) is depicted in the DAG being coordinated using Snakemake. Each box represents an individual Snakemake rule with gray directional arrows indicating the rule as a dependency for another. The “crispy” step in this workflow represents a combination of steps at the per amplicon target level including annotation of variant interrogation window, read realignment (using psnw), variant calling and variant annotation (repair mechanism characterization, etc.).

Tables:

Table S1: Description of all Alt-R S.p. Cas9 guides and amplicons used for sequencing. Included information includes the name of the guide (TargetName), the sequence of the guide (gRNA), and the sequence of the amplicon (AmpliconSequence)

Table S2: Description of all Alt-R A.s. Cas12a Ultra guides and amplicons used for sequencing. Included information includes the name of the guide (TargetName), the sequence of the guide (gRNA), and the sequence of the amplicon (AmpliconSequence)

Table S3: Description of all synthetically modeled rhAmpSeq panels for on/off-target accuracy validation. Included information includes the name of the on/off-target multiplex panel (PanelName), the name of the guide (TargetName), the chromosome of the amplicon/guide (AmpliconChr/GudeChr), the starting position of the amplicon/guide position (AmpliconStart/GuideStart), the end position of the amplicon/guide genomic position (AmpliconStop/GuideStop), and the strand of the guide (GuideStrand). All panels refer to genomic coordinates in the hg38 reference genome.

Table S4: Description of all gBlocks used the titrated DNA pools for analysis of software sensitivity. Included information includes the name of gBlock (name), the sequence of the gBlock (sequence), the variant type (variant_type), the size of the variant mutation (variant_size), the name of the targeted guide (site) and the length of the synthesized gBlock (LEN).

Supplemental Files:

Supplemental File1: Example output files and graphics from the CRISPAltRations software interface for a single amplicon target.
