# Supplementary material for: CRISPAltRations: a validated cloud-based approach for interrogation of double-strand break repair mediated by CRISPR genome editing
Source: Mol Ther Methods Clin Dev. 2021 Apr 2;21:478–91. doi: 10.1016/j.omtm.2021.03.024 (PMC8082044; doi:10.1016/j.omtm.2021.03.024)
Supplement: Data S1. Example output files and graphics from the CRISPRAltRations software interface for a single amplicon target [file mmc6.zip › SupplementalOutputs/metrics.html]

{{pipeline}} {{startDate}} {{targets}}


|  | Group ID | Treated | Total Reads | Percent Edited | Percent Unedited | Percent NHEJ | Percent Perfect HDR | Percent Imperfect HDR | Percent HDR | Percent Other | Percent In Frame | Percent Frameshift | Percent Indels | Percent Insertions | Percent Templated Insertion | Percent GC-Insertion | Percent Deletions | Percent MMEJ | Percent SNP Lines |
| --- | --- | --- | --- | --- | --- | --- | --- | --- | --- | --- | --- | --- | --- | --- | --- | --- | --- | --- | --- |
| Edited | Edited | TRUE | 5078.0 | 97.77 | 2.23 | 96.93 | 0.0 | 0.0 | 0.0 | 0.84 | 30.03 | 69.97 | 96.93 | 22.98 | 72.24 | 1.2 | 74.99 | 15.97 | 8.19 |
