# Supplementary material for: CRISPAltRations: a validated cloud-based approach for interrogation of double-strand break repair mediated by CRISPR genome editing
Source: Mol Ther Methods Clin Dev. 2021 Apr 2;21:478–91. doi: 10.1016/j.omtm.2021.03.024 (PMC8082044; doi:10.1016/j.omtm.2021.03.024)
Supplement: Data S1. Example output files and graphics from the CRISPRAltRations software interface for a single amplicon target [file mmc6.zip › SupplementalOutputs/percentages.html]

{{pipeline}} {{startDate}} {{targets}}


|  | % Edited |  | % Repair Pathways |  | % Frameshift |
| --- | --- | --- | --- | --- | --- |
| Edited | 97.77%2.23% |  | 0.84%0.0%0.0%96.93%2.23% |  | 69.97%30.03% |
|  | UneditedEdited |  | UneditedNHEJPerfectHDRImperfectHDROther |  | InframeFrameshift |
